# Extracts of Immature Orange *(Aurantii fructus immaturus)* and Citrus Unshiu Peel *(Citri unshiu pericarpium)* Induce P-Glycoprotein and Cytochrome P450 3A4 Expression via Upregulation of Pregnane X Receptor

**DOI:** 10.3389/fphar.2017.00084

**Published:** 2017-02-21

**Authors:** Naoto Okada, Aki Murakami, Shiori Urushizaki, Misa Matsuda, Kazuyoshi Kawazoe, Keisuke Ishizawa

**Affiliations:** ^1^Department of Clinical Pharmacy Practice Pedagogy, Institute of Biomedical Sciences, Tokushima University Graduate SchoolTokushima, Japan; ^2^Department of Pharmacy, Tokushima University HospitalTokushima, Japan; ^3^Faculty of Pharmaceutical Sciences, Tokushima UniversityTokushima, Japan; ^4^Department of Clinical Pharmacy, Institute of Biomedical Sciences, Tokushima University Graduate SchoolTokushima, Japan

**Keywords:** Kampo, immature orange, citrus unshiu peel, P-glycoprotein, cytochrome P450 3A4, pregnane X receptor, Drug–Drug Interaction

## Abstract

P-glycoprotein (P-gp) and cytochrome P450 3A4 (CYP3A4) are expressed in the intestine and are associated with drug absorption and metabolism. Pregnane X receptor (PXR) is the key molecule that regulates the expression of P-gp and CYP3A4. Given that PXR activity is regulated by a variety of compounds, it is possible that unknown PXR activators exist among known medicines. Kampo is a Japanese traditional medicine composed of various natural compounds. In particular, immature orange [*Aurantii fructus immaturus* (IO)] and citrus unshiu peel [*Citri unshiu pericarpium* (CP)] are common ingredients of kampo. A previous study reported that kampo containing IO or CP decreased the blood concentration of concomitant drugs via upregulation of CYP3A4 although the mechanism was unclear. Some flavonoids are indicated to alter P-gp and CYP3A4 activity via changes in PXR activity. Because IO and CP include various flavonoids, we speculated that the activity of P-gp and CYP3A4 in the intestine may be altered via changes in PXR activity when IO or CP is administered. We tested this hypothesis by using LS180 intestinal epithelial cells. The ethanol extract of IO contained narirutin and naringin, and that of CP contained narirutin and hesperidin. Ethanol extracts of IO and CP induced P-gp, CYP3A4, and PXR expression. The increase of P-gp and CYP3A4 expression by the IO and CP ethanol extracts was inhibited by ketoconazole, an inhibitor of PXR activation. The ethanol extract of IO and CP decreased the intracellular concentration of digoxin, a P-gp substrate, and this decrease was inhibited by cyclosporine A, a P-gp inhibitor. In contrast, CP, but not IO, stimulated the metabolism of testosterone, a CYP3A4 substrate, and this was inhibited by a CYP3A4 inhibitor. These findings indicate that the ethanol extract of IO and CP increased P-gp and CYP3A4 expression via induction of PXR protein. Moreover, this induction decreased the intracellular substrate concentration.

## Introduction

The intestine functions in the first step of drug absorption. P-glycoprotein (P-gp) and cytochrome P450 3A4 (CYP3A4) are expressed in intestinal epithelial cells and play important roles in this process (Gupta et al., 2008). P-gp is a member of the ATP-binding cassette superfamily of transmembrane proteins and plays a role in the extrusion of a wide variety of substrates (Ambudkar et al., 1999) by intestinal epithelial cells to decrease intracellular substrate concentrations (Harmsen et al., 2010; Sridhar et al., 2014). CYP3A4 is involved in the metabolism of various drugs, and its activation decreases intracellular substrate concentrations (Kolars et al., 1992; Paine et al., 1996; Ruschitzka et al., 2000); specifically, changes in CYP3A4 activity in intestinal epithelial cells affect the bioavailability of drugs. Inhibition of CYP3A4 activity in intestinal epithelial cells increased substrate concentrations in blood (Ohnishi et al., 2000). Therefore, monitoring of changes in P-gp and CYP3A4 activity is important for estimating drug absorption and metabolism in the intestine.

Various compounds modulate P-gp and CYP3A4 levels in the intestine. 1α, 25-Dihydroxyvitamin D_3_ (VD_3_) induces their expression via vitamin D receptor (VDR; Schmiedlin-Ren et al., 2001; Thummel et al., 2001), whereas some drugs increase the activity of the nuclear pregnane X receptor (PXR), which upregulates the expression of P-gp and CYP3A4 (Ma et al., 2005). Intestinal PXR activation induces P-gp and CYP3A4 expression and reduces drug bioavailability by increasing drug efflux and drug metabolism in the intestine (Holmstock et al., 2013; Kawauchi et al., 2014). Given that PXR activity is regulated by a variety of compounds, it is possible that there are unknown drug interactions associated with PXR-induced P-gp or CYP3A4 in intestinal epithelial cells.

Kampo is a traditional Japanese medicine that has been approved by the Ministry of Health, Labor and Welfare of Japan. Kampo medicines are used in combination with western medications or alone as a complementary or alternative therapy. They are a blend of herbal components, which makes it difficult to predict their interaction with concomitantly consumed drugs. Immature orange [*Aurantii fructus immaturus* (IO)] and citrus unshiu peel [*Citri unshiu pericarpium* (CP)] are common ingredients of kampo that are reported to have anti-hypercholesterolemic or orexigenic effects (Yoshie et al., 2004; Hayakawa et al., 2014). However, a previous study reported that kampo containing IO or CP decreased the blood concentration of concomitantly administered CYP3A4 substrates via upregulation of CYP3A4 although the mechanism was unclear (Kinoshita et al., 2011). IO and CP include some flavonoids that have pharmacological effects (Tokunaga et al., 2016). Some flavonoids have been indicated to alter P-gp and CYP3A4 activity via changes in PXR activity (Hiura et al., 2014). Therefore, we speculated that the activity of P-gp and CYP3A4 in the intestine may be altered via changes in PXR activity when IO or CP is administered.

To test this hypothesis, in the present study, we analyzed the effects of IO and CP on P-gp and CYP3A4 expression in LS180 intestinal epithelial cells. Our findings provide insight into potential interactions between kampo containing IO or CP and other drugs via the upregulation of P-gp and CYP3A4 in the intestine as well as a guideline for improving the safety of kampo treatment.

## Materials and Methods

### Materials

IO (#24014051) and CP (#24014541) were purchased from Tsumura (Tokyo, Japan). LS180 cells were purchased from the American Type Culture Collection (Manassas, VA, USA). VD_3_ and 3-(4,5-dimethyl-2-thiazolyl)-2,5-diphenyl-2H-tetrazolium bromide (MTT) were purchased from Sigma-Aldrich (St. Louis, MO, USA). Digoxin was purchased from Tokyo Kagaku (Tokyo, Japan). Cyclosporine A, ketoconazole, rifampicin, and testosterone were purchased from Wako Pure Chemical Industries (Osaka, Japan). Dulbecco’s Modified Eagle Medium (DMEM)+GlutaMAX-1, non-essential amino acids, penicillin/streptomycin, and fetal bovine serum (FBS) were purchased from Life Technologies (Tokyo, Japan), Nacalai Tesque (Tokyo, Japan), DS Pharma Biomedical (Osaka, Japan), and Biowest (Nuaillé, France), respectively. Mouse anti-CYP3A4 (ab3572) and rabbit anti-P-gp (ab3366) antibodies were purchased from Abcam (Cambridge, UK). Rabbit anti-β-actin antibody (#4967) was purchased from Cell Signaling Technology (Beverly, MA, USA). Mouse anti-PXR antibody (SC-48403) was purchased from Santa Cruz Biotechnology (Dallas, TX, USA). Horseradish peroxidase-conjugated sheep anti-mouse IgG and anti-rabbit IgG and enhanced chemiluminescence (ECL) western blotting detection reagent were purchased from GE Healthcare Japan (Tokyo, Japan). The Multi-Drug Resistance Assay and P450-Glo Assay kits were purchased from Cayman Chemical (Ann Arbor, MI, USA) and Promega (Madison, WI, USA), respectively. Architect digoxin and testosterone assay kits were purchased from Abbot Japan (Tokyo, Japan).

### Ethanol Extract Preparation

IO or CP (5 g) was extracted in 50 ml of boiling ethanol for 1 h, followed by centrifugation at 700 × *g* for 5 min, filtration, and concentration using a rotation evaporator. The obtained extracts were dissolved in 55% ethanol and used in this study. The final concentration of each extract was 20 mg of raw material per ml. The final concentration of ethanol was diluted to 0.5% or less; this concentration has been reported to show no cytotoxicity (Timm et al., 2013). These samples were passed through a 0.22-μm membrane filter and used for all experiments.

### Three-Dimensional HPLC Analysis

The ethanol extract (10 μl) of each sample was subjected to three-dimensional high-performance-liquid chromatography (HPLC) analysis to detect the chemical constituents. The HPLC system consisted of a PU-4180 pump, a MD-4015 multiwavelength detector (200–400 nm), and a CO-4060 column oven (JASCO, Tokyo, Japan). The column was a Mightysil RP-18GP S (150 × 4.6 mm i.d.; Kanto Chemical Co., Inc., Tokyo, Japan). The solvents were (A) water/acetonitrile (17:3) and (B) water/acetonitrile (3:2). A linear gradient of 100% A and 0% B changing over 45 min to 0% A and 100% B was applied, and then 0% A and 100% B were continued for 15 min. The flow rate and the column temperature were 1.0 ml per min and 40°C, respectively. Each component was identified by comparing the peak retention times and wavelengths with those of authentic compounds.

### Cell Culture

LS180 cells were cultured in DMEM+GlutaMAX-1 medium containing 10% FBS, 100 U/ml penicillin, 100 μg/ml streptomycin, and 0.1 mM non-essential amino acids at 37°C in an atmosphere of 95% air/5% CO_2_. Cells were cultured to confluence and treated with 100 nM VD_3_, 10 μM rifampicin, or ethanol extracts for 48 h. To inhibit PXR activity, 25 μM ketoconazole was applied 48 h before and during the treatment described above.

### MTT Assay

The MTT assay was performed to assess the cytotoxicity of ethanol extracts. The MTT assay was performed as previously described (Lobner, 2000). Briefly, MTT was added to LS180 cells treated with various regents. After incubation for 2 h, dimethylsulfoxide was added to the cells and the formation of formazan crystals was measured based on the absorbance change using a Varioskan Flash 2.4 microplate reader (Thermo Fisher Scientific, Waltham, MA, USA). Results were normalized to the absorbance of the blank control.

### Immunoblot Analysis of PXR, P-gp, and CYP3A4 Expression

LS180 cells were lysed in lysis buffer consisting of 20 mM Tris-HCl (pH 7.5), 150 mM NaCl, 1 mM Na_2_EDTA, 1 mM EGTA, 1% Triton X-100, 2.5 mM sodium pyrophosphate, 1 mM β-glycerophosphate, 1 mM sodium orthovanadate, and 1 μg/ml leupeptin phenylmethanesulfonyl fluoride on ice and sonicated for 10 s. Cell lysates were centrifuged at 12,000 rpm for 20 min and the supernatant (20 μg protein) was used for sodium dodecyl sulfate (SDS)-polyacrylamide gel electrophoresis. The lysate was mixed with an equal volume of sample buffer containing 50 mM Tris-HCl (pH 8.0), 4% SDS, 2% 2-mercaptoethanol, 10% glycerol, and 0.2% bromophenol blue. After boiling for 5 min, samples were loaded onto gels and separated, and the proteins were transferred to nitrocellulose membranes. Following each antibody treatment (dilution of 1:1000 for all antibodies), protein bands were visualized using the ECL kit. ImageJ v.1.37 software (National Institutes of Health, Bethesda, MD, USA) was used to measure signal intensity, which was normalized to that of β-actin.

### P-gp Activity Assay

P-glycoprotein transport activity was determined to assess the effect of induction of P-pg protein on P-gp activity in intestinal epithelial cells. Cells were treated with VD_3_ or each ethanol extract, and these were washed out after 48 h of incubation. Then, cells were treated with 10 μM cyclosporine A for 15 min to inhibit P-gp activity. After the cyclosporine A was washed out, P-gp activity was measured using the Multi-Drug Resistance Assay kit according to the manufacturer’s instructions. Phosphate buffer containing 2 μM calcein-AM was added to the sample, followed by incubation for 30 min. Calcein fluorescence was measured using a microplate reader.

### CYP3A4 Activity Assay

Cytochrome P450 3A4 metabolism was evaluated to assess the effect of induction of CYP3A4 protein on CYP3A4 activity in intestinal epithelial cells. Each reagent was added to the cultured cells and washed out after 48 h of incubation. Then, 10 mM ketoconazole was applied for 15 min to inhibit CYP3A4 activity. After the ketoconazole was washed out, CYP3A4 activity was measured using the P450-Glo assay kit according to the manufacturer’s instructions. Culture medium containing 3 μM luciferin-IPA, a highly specific CYP3A4 substrate, was added to the cells for 1 h, followed by addition of luciferin detection reagent for 30 min. Luciferin fluorescence was measured using a microplate reader.

### Measurement of Intracellular Drug Concentration

After treatment with various reagents for 48 h, the reagents were washed out, and the treated cells were used for experiments. The treated cells were incubated with 0.1 μM digoxin or 0.4 μM testosterone for 90 min. To inhibit P-gp and CYP3A4 activity, cells were pretreated with 10 μM cyclosporine A or 10 mM ketoconazole for 30 min before treatment with digoxin or testosterone, respectively. After the removal of culture medium and washing out of any residual reagent, the cells were sonicated. The lysates were centrifuged at 12,000 rpm for 10 min, and supernatants were used for the assay. Digoxin and testosterone concentrations were measured by chemiluminescent immunoassay using Architect digoxin and testosterone assay kits, respectively. The amount of protein in each sample was measured by the Bradford method and results were normalized to the amount of total protein.

### Data Analysis

Each experiment was performed three times, and results are expressed as the mean ± SD. One-way analysis of variance was used to compare group means, and differences between groups were analyzed using the Bonferroni method. All recorded P values were two-sided, and *P* < 0.05 was considered statistically significant.

## Results

### Three-Dimensional High-Performance Liquid Chromatography

**FIGURE 1:**
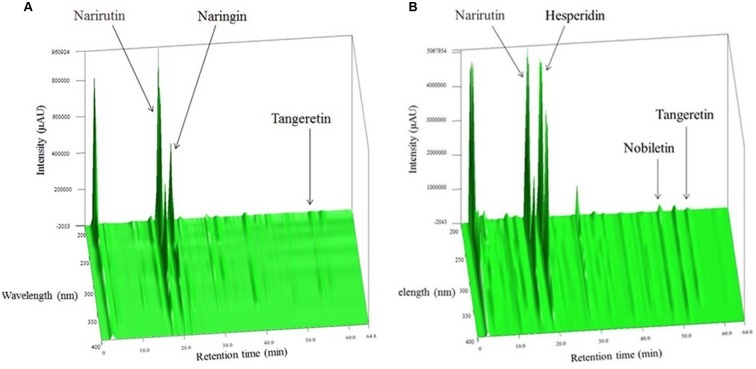
**Three-dimensional HPLC analysis of** **(A)** IO and **(B)** CP ethanol extracts.

The components of IO or CP ethanol extracts were analyzed by three-dimensional high-performance liquid chromatography (**Figures 1A,B**). Narirutin and naringin were the main compounds in IO ethanol extracts, and narirutin and hesperidin were the main components in CP ethanol extracts. Almost no other compounds were detected in each extract.

### Cytotoxicity of IO and CP Ethanol Extracts

**FIGURE 2:**
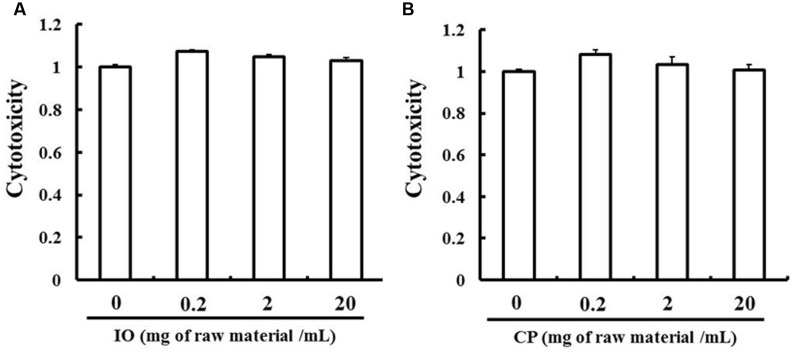
**Evaluation of IO** **(A)** and CP **(B)** cytotoxicity. Results represent the mean ± SD of three separate measurements and are expressed relative to a concentration of 0 mg of raw material per ml; IO, Kijitsu; CP, Chinpi.

The MTT assay was used to assess the cytotoxicity of the ethanol extracts to LS180 cells. The extracts were not toxic up to a concentration of 20 mg of raw material per ml (**Figure 2**). Based on these results, we used IO and CP ethanol extracts at a concentration of 20 mg of raw material per ml in all experiments.

### PXR-Induced Upregulation of P-gp and CYP3A4 in LS180 Cells Treated with IO or CP

**FIGURE 3:**
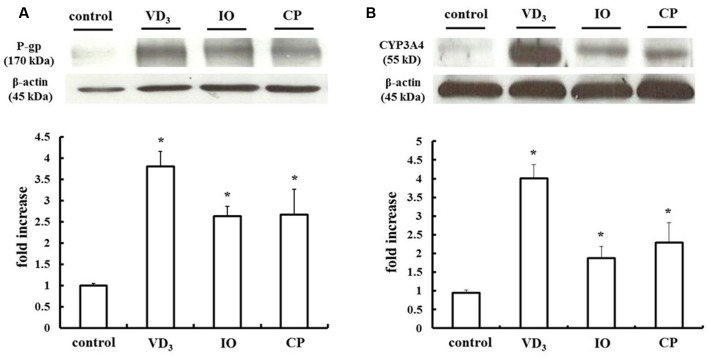
**Effect of IO and CP on protein expression of** **(A)** P-gp and **(B)** CYP3A4. Bar graphs show the average signal strength. Results represent the mean ± SD of three separate measurements and are expressed relative to the control. ^∗^*P* < 0.01. CYP3A4, cytochrome P450 3A4; IO, Kijitsu; P-gp, p-glycoprotein; CP, Chinpi; VD_3_, 1α,25-dihydroxyvitamin D_3_.

**FIGURE 4:**
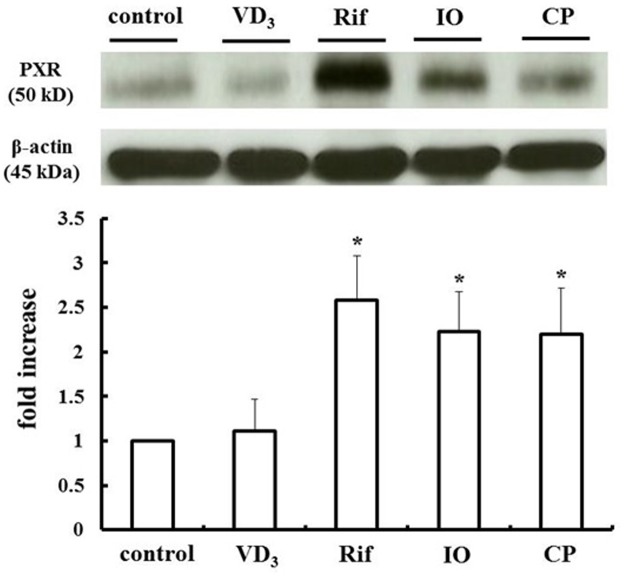
**IO and CP upregulate PXR protein.** Bar graphs show the average signal strength. Results represent the mean ± SD of three separate measurements and are expressed relative to the control. ^∗^*P* < 0.01. IO, Kijitsu; PXR, pregnane X receptor; Rif, rifampicin; CP, Chinpi.

**FIGURE 5:**
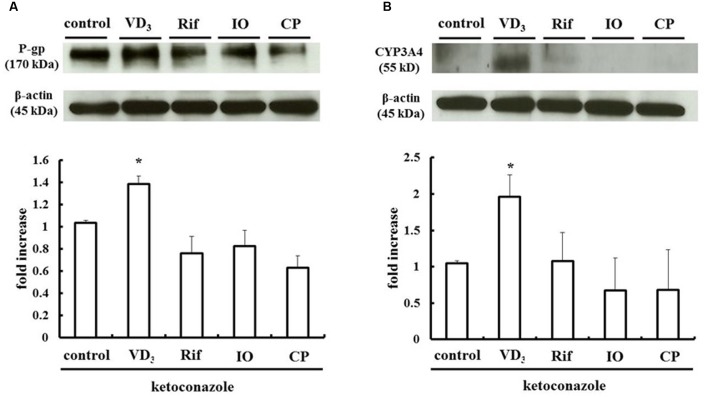
**Ketoconazole inhibits the upregulation of** **(A)** P-gp and **(B)** CYP3A4 by IO and CP. Ketoconazole (25 μM) was pretreated 48 h before and during IO or CP treatment to inhibit PXR activity. Bar graphs show the average signal strength. The results represent the mean ± SD of three separate measurements and are expressed relative to the control. ^∗^*P* < 0.05. CYP3A4, cytochrome P450 3A4; IO, Kijitsu; P-gp, p-glycoprotein; Rif, rifampicin; CP, Chinpi; VD_3_, 1α,25-dihydroxyvitamin D_3_.

We measured changes in P-gp and CYP3A4 protein expression in LS180 cells treated with IO or CP ethanol extracts. As previously reported, P-gp and CYP3A4 were upregulated by VD_3_ treatment (Inami et al., 2015); the expression of these proteins was also increased by approximately 2.0- and 2.5-fold by IO and CP treatment, respectively, relative to controls (**Figures 3A,B**). To clarify the mechanism, the protein level of the PXR, a nuclear receptor regulating the expression of P-gp and CYP3A4, was determined (**Figure 4**). VD_3_ has been reported to induce P-gp and CYP3A4 protein expression in a PXR-independent manner (Schmiedlin-Ren et al., 2001). We found that the PXR protein level was unaltered by VD_3_ treatment in LS180 cells. In contrast, it was increased by treatment with IO or CP ethanol extract, as well as treatment with rifampicin, a PXR inducer (Jiang et al., 2009). P-gp and CYP3A4 protein levels were increased by VD_3_ treatment despite pretreatment with ketoconazole, a PXR activity inhibitor (**Figures 5A,B**). In contrast, ketoconazole abrogated the increases in P-gp and CYP3A4 protein expression induced by rifampicin, consistent with previous reports (Huang et al., 2007; Svecova et al., 2008), as well as the increases induced by IO or CP ethanol extracts. These results indicate that IO and CP induce P-gp and CYP3A4 expression via upregulation of PXR in LS180 cells.

### Pharmacokinetic Changes Induced by IO and CP

**FIGURE 6:**
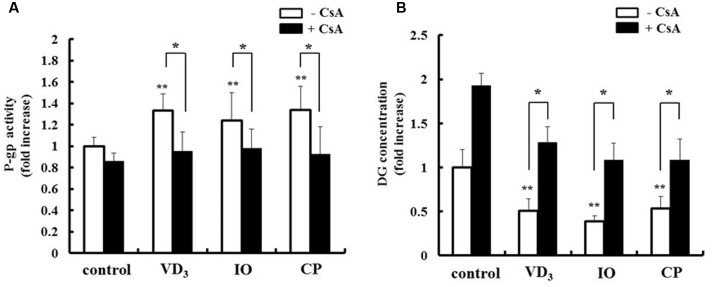
**Effects of IO and CP on** **(A)** P-gp activity and **(B)** intracellular concentrations of the P-gp substrate digoxin. Cells were pretreated with cyclosporine A (10 μM) for 15 min to inhibit P-gp activity before addition of DG. Results represent the mean ± SD of three separate measurements and are expressed relative to the control without cyclosporine A. ^∗^*P* < 0.01, ^∗∗^*P* < 0.01 vs. control. CsA, cyclosporine A; DG, digoxin; IO, Kijitsu, P-gp, p-glycoprotein; CP, Chinpi; VD_3_, 1α,25-dihydroxyvitamin D_3_.

**FIGURE 7:**
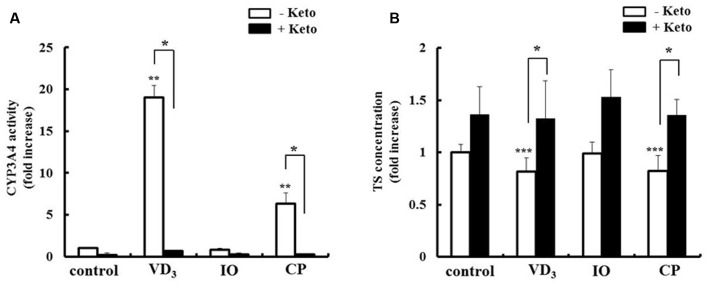
**Effects of IO and CP on** **(A)** CYP3A4 activity and **(B)** intracellular concentrations of the CYP3A4 substrate testosterone. Cells were pretreated with ketoconazole (10 mM) for 15 min to inhibit CYP3A4 activity before addition of TS. Results represent the mean ± SD of three separate measurements and are expressed relative to the control without ketoconazole. ^∗^*P* < 0.01, ^∗∗^*P* < 0.01 vs. control, ^∗∗∗^*P* < 0.05 vs. control. CYP3A4, cytochrome P450 3A4, keto, ketoconazole; IO, Kijitsu, CP, Chinpi; TS, testosterone; VD_3_, 1α,25-dihydroxyvitamin D_3_.

We analyzed the pharmacokinetic effects of P-gp and CYP3A4 upregulation in LS180 cells. The drug extrusion activity of P-gp was increased by extract treatment (**Figure 6A**). Moreover, the intracellular concentration of the P-gp substrate digoxin decreased in the presence of both IO and CP (**Figure 6B**), and this effect was abrogated by treatment with the P-gp inhibitor cyclosporine A. CP but not IO extract increased the metabolic activity of CYP3A4 (**Figure 7A**). The intracellular concentration of testosterone, a CYP3A4 substrate, was decreased by CP but not IO treatment (**Figure 7B**), and this effect was inhibited by short-term treatment with the CYP3A4 inhibitor ketoconazole.

## Discussion

Our study demonstrated that IO and CP ethanol extracts increased P-gp and CYP3A4 protein levels in LS180 cells, which was associated with increased PXR expression. While CP decreased intracellular substrate concentrations of both P-gp and CYP3A4, IO acted only on the former.

The expression of P-gp and CYP3A4 is mediated by various factors; VD_3_ has been shown to increase P-gp and CYP3A4 levels in a VDR-dependent manner (Schmiedlin-Ren et al., 2001), whereas rifampicin exerts a similar effect via PXR induction and activation (Healan-Greenberg et al., 2008; Svecova et al., 2008). In our study, IO and CP ethanol extracts increased PXR expression. Moreover, the upregulation of P-gp and CYP3A4 by IO and CP was inhibited by pretreatment with ketoconazole, a PXR activity inhibitor. These results indicate that IO and CP ethanol extract increased P-gp and CYP3A4 expression via upregulation of PXR. Moreover, P-gp and CYP3A4 induced by IO and CP showed adequate extrusion or metabolism ability (**Figures 6** and **7**). This indicated that these proteins were expressed at appropriate site and folded adequately. Constitutive androstane receptor 1 (CAR1) is an important transcription receptor for inducing P-gp and CYP3A4 expression (Goodwin et al., 2002). However, a previous study demonstrated that CAR1 transcripts were not expressed in LS 180 cells (Gupta et al., 2008). These data support our results that induction of P-gp and CYP3A4 protein by IO and CP in LS180 cells is mediated by PXR.

Pregnane X receptor is a key protein controlling drug absorption and metabolism in the intestine via regulation of P-gp and CYP3A4 activity. Therefore, agents that alter PXR activity are expected to affect the absorption and metabolism of concomitantly administered drugs (Qiao et al., 2013). PXR activity is altered in at least one of the following ways: (i) activation of PXR function by PXR ligands or (ii) induction of PXR protein expression (Yang et al., 2011). Most P-gp and CYP3A4 inducers are PXR ligands and activate PXR function, and a few directly stimulate PXR protein expression. It has been reported that hesperetin, which is one of the main flavonoids in CP, did not affect PXR-dependent transcriptional activity in a luciferase reporter assay (Satsu et al., 2008). Moreover, there are no reports showing that narirutin, the main flavonoid in each extract in the present study, affects PXR-dependent transcriptional activity. Therefore, in our study, we focused on the PXR expression change induced by IO and CP. We speculated that narirutin was a key compound that upregulated PXR expression, because narirutin was a common compound in each extract. Alternatively, the flavonoids contained in each ingredient might cooperate to increase the expression of PXR. Although the detailed mechanism by which narirutin induces PXR expression remains unclear, this is the first report demonstrating upregulation of PXR by components of kampo.

IO and CP ethanol extracts induced P-gp excretion, indicating that kampo containing IO or CP may alter the absorption of P-gp substrates in the intestine via upregulation of P-gp protein. We also showed that CP induced metabolism and decreased the intracellular concentration of CYP3A4 substrate, whereas IO did not have this effect despite inducing upregulation of CYP3A4 protein. These suggested that bioavailability of the substrates may decrease via the upregulation of P-gp and CYP3A4 in intestinal epithelial cells when IO or CP is administered. Our results provide novel evidence for a mechanism of interaction between kampo including IO and CP and other drugs via the upregulation of P-gp and CYP3A4. A previous study revealed that naringin and hesperidin had inhibitory effects on CYP3A4 (Ho et al., 2001). The effect of naringin on the inhibition of CYP3A4 might have been clearly observed because of the difference in the content of the CYP3A4 inhibitor flavonoid in each extract. Our results highlight differences in the pharmacokinetic changes induced by IO and CP.

Flavonoids included in IO and CP have various pharmacological effects (Tokunaga et al., 2016). Ethanol extracts of crude drugs include both hydrophilic and hydrophobic flavonoids. In our study, the water extract of IO and CP increased P-gp and CYP3A4 protein expression, but this effect was weaker than that of ethanol extracts (data not shown). A previous study revealed that narirutin could be extracted from citrus fruits with the highest yield when using 50–70% ethanol solution; the amount of narirutin extracted by water was half of that extracted using 50–70% ethanol solution (Miyake, 2006). The lower amount of narirutin in the water extract of IO and CP might be the reason why the water extract of IO and CP had a weaker effect on the upregulation of P-gp and CYP3A4 protein expression in the present study.

IO and CP are used as a traditional medicine for treatment of abdominal symptoms. Daisaiko-to, which contains IO, has anti-hypercholesterolemic effects (Yoshie et al., 2004); rikkunshi-to, which contains CP, has been reported to exert orexigenic effects by stimulating ghrelin secretion (Hayakawa et al., 2014). Thus, kampo containing IO and CP has various clinical applications. However, based on our results, the possibility of pharmacokinetic changes with concomitant drug use should be considered when administering kampo containing IO and CP as a treatment.

We examined changes in intracellular drug concentration using LS180 cells, which are a suitable intestinal epithelial cell model to analyze interactions between drugs and epithelial cells (Gupta et al., 2008; Lau et al., 2013), although *in vivo* studies are needed to confirm our findings. A previous study demonstrated that hochuekki-to containing CP increased CYP3A4 expression in the intestine and decreased the area under the curve for the CYP3A4 substrate nifedipine (Kinoshita et al., 2011). This indicated that that induction of P-gp and CYP3A4 protein via upregulation of PXR in intestinal epithelial cells may occur *in vivo*. However, it was recently reported that daisaiko-to containing IO increased the area under the curve for nifedipine *in vivo* (He et al., 2014); although the underlying mechanism is unclear, this finding suggests that the activity and expression of P-gp and CYP3A4 may differ according kampo formulations. Therefore, a more detailed analysis of different types of kampo is needed to broaden its clinical applicability.

## Conclusion

Our study demonstrated that ethanol extracts of IO and CP induced P-gp and CYP3A4 expression via upregulation of PXR and decreased intracellular substrate concentrations, thereby potentially perturbing drug absorption and metabolism. Although further analyses are needed to confirm our results, these findings provide information that can inform the safe use of kampo.

## Author Contributions

NO, AM, KK, and KI conceived of and designed the experiments. AM, SU, and MM performed the experiments. NO drafted the manuscript. All authors read and approved the final manuscript.

## Conflict of Interest Statement

The authors declare that the research was conducted in the absence of any commercial or financial relationships that could be construed as a potential conflict of interest.
